# Optimizing Clinical Diabetes Diagnosis through Generative Adversarial Networks: Evaluation and Validation

**DOI:** 10.3390/diseases11040134

**Published:** 2023-09-30

**Authors:** Antonio García-Domínguez, Carlos E. Galván-Tejada, Rafael Magallanes-Quintanar, Miguel Cruz, Irma Gonzalez-Curiel, J. Rubén Delgado-Contreras, Manuel A. Soto-Murillo, José M. Celaya-Padilla, Jorge I. Galván-Tejada

**Affiliations:** 1 Unidad Académica de Ingeniería Eléctrica, Universidad Autónoma de Zacatecas, Jardín Juárez 147, Centro, Zacatecas 98000, Mexico; antonio.garcia@uaz.edu.mx (A.G.-D.); tiquis@uaz.edu.mx (R.M.-Q.); ruben.delgado@uaz.edu.mx (J.R.D.-C.); alejandro.somu@uaz.edu.mx (M.A.S.-M.); jose.celaya@uaz.edu.mx (J.M.C.-P.); gatejo@uaz.edu.mx (J.I.G.-T.); 2 Medical Research Unit in Biochemestry, National Medical Center Siglo XXI, IMSS, Mexico City 06720, Mexico; mcruzl@yahoo.com; 3 Unidad Académica de Ciencias Químicas, Universidad Autónoma de Zacatecas, Jardín Juarez 147, Centro, Zacatecas 98000, Mexico; irmacuriel@uaz.edu.mx

**Keywords:** generative networks, Type 2 Diabetes, predictive modeling, clinical data

## Abstract

The escalating prevalence of Type 2 Diabetes (T2D) represents a substantial burden on global healthcare systems, especially in regions such as Mexico. Existing diagnostic techniques, although effective, often require invasive procedures and labor-intensive efforts. The promise of artificial intelligence and data science for streamlining and enhancing T2D diagnosis is well-recognized; however, these advancements are frequently constrained by the limited availability of comprehensive patient datasets. To mitigate this challenge, the present study investigated the efficacy of Generative Adversarial Networks (GANs) for augmenting existing T2D patient data, with a focus on a Mexican cohort. The researchers utilized a dataset of 1019 Mexican nationals, divided into 499 non-diabetic controls and 520 diabetic cases. GANs were applied to create synthetic patient profiles, which were subsequently used to train a Random Forest (RF) classification model. The study’s findings revealed a notable improvement in the model’s diagnostic accuracy, validating the utility of GAN-based data augmentation in a clinical context. The results bear significant implications for enhancing the robustness and reliability of Machine Learning tools in T2D diagnosis and management, offering a pathway toward more timely and effective patient care.

## 1. Introduction

Diabetes Mellitus (DM) encompasses a range of metabolic disorders defined by hyperglycemia resulting from defects in insulin secretion, insulin action, or both [1]. This hyperglycemia can lead to a range of acute and chronic complications including diabetic ketoacidosis, cardiovascular diseases, and microvascular conditions such as retinopathy and nephropathy. DM’s multifactorial etiology has made it a global public health concern due to its rising prevalence, negative impact on quality of life, potential for severe complications, and the complexity of treatment regimens [2].

Type 2 Diabetes (T2D), which primarily originates from insulin resistance, is responsible for over 90% of DM cases and manifests as systemic organ dysfunction, including, but not limited to cardiovascular, renal, and neurological systems [3]. Early detection and management of T2D are pivotal for reducing the risk of complications and improving outcomes, thus providing clinicians with data for more-effective treatment and preventative strategies.

In Mexico, T2D is increasingly affecting public health and significantly reducing the quality of life for those afflicted. The burgeoning fields of artificial intelligence (AI) and data science have shown promise in biomedical research and offer potential solutions to healthcare challenges such as these [2,4]. However, the effective deployment of these technologies often faces hurdles such as data sensitivity, the high costs of comprehensive data collection, and multidisciplinary collaboration. Challenges also exist in obtaining patient consent and standardizing data across various demographic groups and healthcare sectors.

In this context, our study aimed to leverage GANs for generating synthetic T2D patient data, integrating both clinical and paraclinical metrics, within a Mexican dataset. These synthetic data were used to assess the performance of a Random Forest (RF) classification model trained on both real and synthetic data. The evaluation included a comparison of the model’s performance with the outcomes reported by García et al. [5] for the original dataset.

The rest of the paper is structured as follows: Section 2 details the materials and methods, describing the original dataset and the AI techniques used. Section 3 presents the experimental design and results, and Section 4 offers a discussion on the medical applicability of this study, concluding with key findings and future directions for this line of research.

## 2. Related Work

The landscape of biomedical research has witnessed remarkable transformations with the growing integration of artificial intelligence (AI) and data science methodologies. These advancements have been particularly pronounced in the application of Generative Adversarial Networks (GANs) for generating synthetic medical data, a trend that holds significant potential for addressing various healthcare challenges. Several studies in this domain merit closer examination.

Skandarani et al. [6] made a substantial contribution by focusing on the generation of synthetic medical images using GANs. Their work showcased the ability of GANs to generate high-fidelity images, which can be invaluable for medical imaging research. However, while their approach demonstrated impressive visual realism, it often fell short in terms of producing structured clinical and paraclinical data, which is crucial for comprehensive healthcare research. This limitation underscores the need for a more-holistic approach when generating synthetic medical datasets.

Gonzalez-Abril et al. [7] embarked on generating synthetic lung cancer patient data, providing critical insights into the potential applications of synthetic datasets for medical research. Their work underscores the utility of GANs in generating patient profiles, yet it primarily focused on a specific medical condition. This narrow scope limits the broader applicability of their findings to a wider range of healthcare research scenarios, including diabetes-related studies.

In a comprehensive review, Jiang et al. [8] offered a thorough examination of deep learning techniques in medical-image-based cancer diagnosis. While their work provided a valuable overview of the field’s progress, it predominantly addressed the imaging domain and did not delve deeply into the generation of structured clinical and paraclinical data, a key aspect of holistic healthcare research.

Turning our attention specifically to diabetes research, Zhu et al. [9] introduced “GluGAN”, a GAN-based model tailored to generating personalized glucose time-series data. Their work exemplified the potential of GANs in generating longitudinal patient data. Nonetheless, it primarily focused on glucose data, leaving room for expanding the scope to encompass a wider array of clinical and paraclinical metrics relevant to Type 2 Diabetes (T2D) research.

Furthermore, Vidal et al. [10] applied image-to-image GANs to generate Optical Coherence Tomography (OCT) images, contributing significantly to the field of diabetic retinopathy research. This work highlighted the potential of GANs in generating medical images relevant to diabetes complications. However, the scope remained limited to specific imaging modalities, and the generation of structured clinical data was not explored.

Tanaka et al. [11] proposed the use of Generative Adversarial Networks (GANs) for generating artificial training data in Machine Learning tasks. Their focus was on generating synthetic data, which can be highly valuable in situations such as imbalanced datasets, serving a role similar to the Synthetic Minority Over-sampling Technique (SMOTE) or Adaptive Synthetic Sampling (ADASYN). Furthermore, they highlighted its utility when data contain sensitive information, such as medical data, and it is desirable to minimize the use of the original dataset. In their experiments, they evaluated the performance of a Decision Tree-based classifier trained using data generated by GANs and compared it with a classifier trained on the original dataset. Their results showed that the GAN-based classifier achieved similar and, in some cases, even better accuracy and recall compared to a classifier trained on the original dataset.

In light of the above, while these previous studies showcased the increasing relevance of GANs in medical research and diabetes-related domains, they also underscored certain limitations, primarily centered on the comprehensiveness of synthetic data generation. Notably, the generation of structured clinical and paraclinical metrics relevant to T2D research remains an underexplored area. This study aimed to bridge this gap by leveraging GANs to synthesize comprehensive T2D patient data, encompassing a wide range of clinical and paraclinical metrics. Furthermore, a rigorous assessment of the synthetic data’s effectiveness was conducted through the training of a Random Forest (RF) classification model. This approach was intended to offer a comprehensive viewpoint on the utilization of GANs in diabetes research, with the ultimate goal of contributing to the advancement of AI techniques in healthcare.

## 3. Material and Methods

This section provides a comprehensive description of the dataset used for training the Generative Adversarial Network (GAN) proposed in this study. It then delves into the details of the GAN itself, followed by an overview of the Machine Learning algorithm utilized for testing this proposition.

### 3.1. Original Dataset Description

The data utilized in this study were graciously provided by the Unidad de Investigación Médica en Bioquímica at the Centro Médico Nacional Siglo XXI, Instituto Mexicano del Seguro Social (IMSS). All participating patients, all of whom were Mexican nationals, duly signed an informed consent form prior to the commencement of the study. The study protocol, adhering to the Helsinki Declaration’s ethical standards, secured approval from the Ethics Committee of the IMSS, Approval Number R-2011-785-018. The collected data, summarized in Table 1, encompassed basic demographic information and pertinent laboratory indicators of the patients. These data points were subsequently extracted for each participant and employed for further analysis.

The dataset utilized in this study included data from 1019 individuals, all of whom are Mexican nationals. This dataset consisted of 499 non-diabetic patients, serving as the control group, and 520 patients who have been diagnosed with diabetes, forming the case group. The age of the participants fell within the range of 35 to 65 years. In terms of sex distribution, the sample was nearly balanced, with 502 participants being female and 517 being male.

### 3.2. Generative Adversarial Networks

Generative Adversarial Networks (GANs) have emerged as a powerful class of artificial intelligence models designed to generate synthetic data that closely approximate real data distributions. Introduced by Goodfellow et al. [12] in 2014, GANs have garnered significant attention across various domains due to their remarkable capacity to create data instances that exhibit similar statistical properties as the original dataset. In the context of this research, GANs play a pivotal role in augmenting the available diabetes patient data.

#### 3.2.1. GAN Architecture

The GAN architecture comprises two primary components: the Generator and the Discriminator. Understanding these components is crucial for grasping the essence of GANs:

- Generator: The Generator serves as the data generator within GANs. It learns to map random noise vectors sampled from a latent space to the data space of interest, which, in this case, is the domain of diabetes patient data. Throughout the training process, the Generator continually refines its output, aiming to produce data samples that become increasingly indistinguishable from authentic patient data. The architecture of the Generator is critical to the success of GANs, as its primary objective is to deceive the Discriminator and generate synthetic data that closely resemble real patient data. Deep learning techniques, such as convolutional layers and recurrent networks, are often employed within the Generator to capture the underlying distribution of real data and generate compelling examples that enrich the original dataset.
- Discriminator: The Discriminator operates as a binary classifier within GANs. It is tasked with distinguishing between instances of real patient data from the original dataset and synthetic data generated by the Generator. This component plays a pivotal role in the training of GANs, as it is trained to discern between authentic and fake data. As the training process advances, the Discriminator enhances its capacity to differentiate between the two categories, driving the Generator to produce data that are increasingly convincing.

#### 3.2.2. Training GANs

GANs employ a unique approach to enhance the quality of the generated data. They achieve this by instigating a competitive learning process between the Generator and the Discriminator:

- Generator training: The Generator is initially untrained and starts by producing random synthetic data samples. As training progresses, the Generator fine-tunes its parameters through gradient-based optimization techniques, such as Stochastic Gradient Descent (SGD) or Adam. The objective is to generate synthetic data that become progressively indistinguishable from real patient data.
- Discriminator training: The Discriminator begins with random weights and gradually adapts to its task of distinguishing real from synthetic data. Similar to the Generator, it undergoes training iterations and optimizes its parameters to improve its classification accuracy.
- Adversarial training: The core principle underlying GANs revolves around the adversarial training procedure. The Generator and the Discriminator are locked in a continuous contest. The Generator aims to create synthetic data that become increasingly indistinguishable from real patient data, while the Discriminator strives to distinguish between real and synthetic data accurately.

#### 3.2.3. Validation of GAN-Generated Data

The validation of GAN-generated data is a crucial step to ensure their quality and suitability for downstream tasks. In this study, the quality and utility of the synthetic diabetes patient data generated by GANs were assessed through comprehensive validation processes:

- Classification model evaluation: To assess the effectiveness of GAN-generated data, a classification model was trained using both real and synthetic data. The performance of this model was evaluated to determine how well it could classify patients into distinct categories based on their diabetes status. When the model’s behavior with synthetic data closely resembles its behavior with real data, this indicates that the generated data are of high quality and effectively capture the essential features of real patient data.
- Comprehensive feature analysis: Additionally, a comprehensive analysis of the features present in the synthetic data was conducted to ensure that important attributes relevant to diabetes research were faithfully represented. This analysis involved statistical comparisons and feature distribution assessments between real and synthetic data.

The incorporation of GAN-generated data into the research process served to augment the quantity and diversity of patient data, addressing data scarcity issues and enhancing the comprehensiveness of the study.

### 3.3. Machine Learning Classifier Algorithms

Machine Learning algorithms play a pivotal role in the field of data science, enabling the development of predictive models from labeled datasets. These algorithms are designed to learn intricate patterns and relationships within the data, allowing them to make accurate predictions on previously unseen instances. In the context of medical data analysis, classifier algorithms take on particular significance, as they facilitate the classification of patients into distinct categories based on their health conditions, ultimately aiding in early diagnosis and treatment planning for various diseases, including diabetes.

One of the standout algorithms among the diverse range of classifier options is the Random Forests algorithm. Developed based on Decision Trees [13], Random Forests have proven to be a powerful and versatile tool for handling classification tasks in medical research.

**Ensemble learning and Decision Trees:**

Random Forests, a form of ensemble learning, differ significantly from traditional single Decision Tree models. They harness the collective wisdom of multiple Decision Trees, improving predictive accuracy and robustness.

Ensemble learning entails constructing a multitude of Decision Trees, each of which operates on a random subset of features and bootstrapped data samples. This process introduces diversity among the individual trees, making them less correlated. As a result, Random Forests are less prone to overfitting, a common challenge in Machine Learning. The final prediction from a Random Forest is derived through a voting mechanism, where each tree contributes its output to the overall decision.

**Enhanced handling of high-dimensional data:**

Medical datasets frequently exhibit high dimensionality, characterized by numerous features and variables. Random Forests excel in this scenario as they can efficiently handle datasets with a large number of features. This characteristic is particularly valuable when dealing with medical data, which often present complex and heterogeneous characteristics. Moreover, Random Forests have the ability to cope with data that can be both numerical and categorical, without requiring extensive preprocessing [13,14].

**Interpretability and feature importance:**

Another key advantage of Random Forests lies in their capacity to provide an assessment of feature importance. This means we can identify which of the features contribute most significantly to the classification process in the context of medical diagnosis [15]. This feature is crucial as it provides valuable insights for medical professionals in understanding the variables that have a significant impact on model predictions.

In this study, Random Forests were employed as the primary classifier for evaluating the performance of the proposed GAN-generated synthetic data. This choice was driven by several factors. Firstly, Random Forests’ capacity to efficiently leverage information from diverse features aligns with the study’s goal of improving diabetes classification results. Additionally, the interpretability of Random Forests contributes to a comprehensive understanding of the classification process, providing valuable insights for healthcare professionals [15].

## 4. Experiments and Results

The experimental process was designed to evaluate the efficacy of the proposed Generative Adversarial Network (GAN) for generating synthetic medical data, which was then used for diabetes classification. This section provides an in-depth overview of the experiments, which were divided into three major components: GAN configuration and training, data augmentation, and diabetes classification performance.

### 4.1. GAN Configuration and Training

The initial phase of the research involved defining a well-structured GAN, with a keen focus on selecting appropriate parameter values for its components. The architecture of the Generator and Discriminator, along with their respective layer sizes, activation functions, and learning rates, was meticulously defined to optimize the performance of the GAN. The final configuration was strategically chosen to strike an optimal balance between model complexity and generative capacity, thus ensuring the generation of high-quality synthetic data. Table 2 provides the details of the selected GAN configuration.

### 4.2. Data Augmentation

The process of augmenting the dataset commenced with the application of the GAN on the original data. Utilizing the GAN’s generative capabilities, synthetic data samples were meticulously generated to complement the existing real-world instances. The seamless fusion of the generated synthetic data with the original dataset resulted in an amplified dataset that now encompassed a harmonious combination of real and synthetic data points.

The augmentation process adhered to the commonly accepted practice of achieving a 30% increase in the dataset size through synthetic data generation [16]. This proportion is considered optimal as it strikes a balance between introducing diversity into the dataset and maintaining its integrity by not excessively skewing the composition with synthetic data. The expanded dataset thus represents a more-comprehensive and -diverse collection of diabetes data, providing a solid foundation for a robust evaluation of the Random Forests classifier’s performance. Table 3 details the size of both the original and the expanded datasets.

In this work, an expansion of the existing dataset was performed by generating an additional 306 data samples, equating to a 30% increase in the dataset size. It is crucial to emphasize that the allocation of these synthetic data samples into the categories of cases (individuals with diabetes) or controls (individuals without diabetes) was determined through a randomized process. This deliberate randomization was implemented to ensure the incorporation of diversity and to maintain the representativeness of the synthetic samples within the dataset.

### 4.3. Model Training and Performance Evaluation

**Figure 1:**
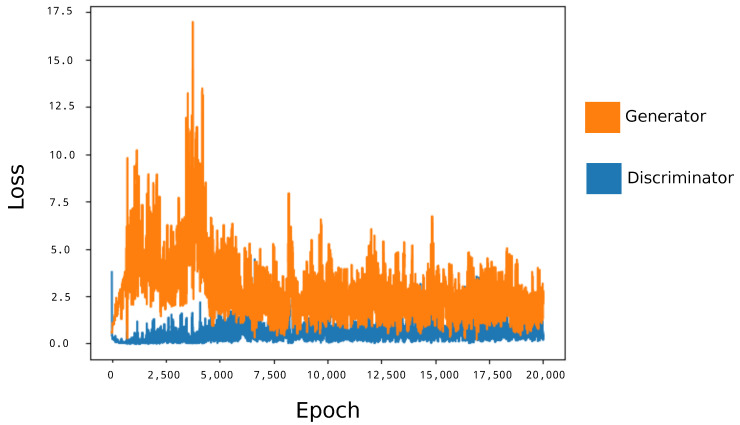
Loss of Generator and Discriminator in the GAN training process: The graph illustrates the evolution of the loss values for both the Generator and the Discriminator components over the training iterations, offering insights into model convergence and stability.

Figure 1 illustrates the loss curves for the Generator and Discriminator during the GAN training process. These curves provide invaluable insights into the convergence and performance of the GAN model, elucidating the learning dynamics and stability achieved during the training iterations.

The final stage of the experimental process involved the development of a classification model using the Random Forests algorithm, leveraging the augmented dataset created by the GAN. The primary objective of this stage was to rigorously evaluate the quality and utility of the synthetic data generated by the GAN. By employing Random Forests, known for its effectiveness in handling diverse features and capturing complex relationships, the experiment aimed to demonstrate how the enlarged dataset could contribute to enhancing the performance and robustness of the diabetes classification task. Through this methodology, the viability of using GAN-generated synthetic data as a valuable asset for improving the accuracy and generalizability of the diabetes classification model was assessed.

Table 4 presents the performance metrics of the Random Forests classification model, as recommended by García et al. [5]. These metrics offer a comprehensive understanding of the effectiveness and reliability of the classifier when trained on the expanded dataset, thereby substantiating the potential advantages of utilizing GAN-generated synthetic data for improving the model’s predictive capabilities.

Table 5 displays the evaluation outcomes of the Random Forests classification model presented by García et al. [5]. These outcomes serve as a benchmark for comparison with the results attained in the current research, where the classification model was assessed using the augmented dataset generated by GANs. This comparative analysis underscores the contribution of GAN-generated synthetic data in enhancing the model’s performance and predictive efficacy.

In addition to comparing our results with the findings of García et al. [5], we also benchmarked our study against the work of Tanaka et al. [11]. In comparison to Tanaka et al.’s findings, our study demonstrated notable advancements in diabetes classification. While Tanaka et al. achieved a test accuracy of 0.748 using GAN-generated synthetic data, our approach surpassed this accuracy with a test accuracy of 0.96 (Table 4). Furthermore, Tanaka et al.’s work focused on addressing imbalanced datasets and avoiding direct use of sensitive medical data, primarily using Decision Trees for classification. In contrast, our study specifically targeted diabetes classification and employed Random Forests, achieving not only a higher accuracy, but also improved sensitivity, specificity, F1-score, and precision (Table 4). These results underscore the substantial enhancement in diabetes classification performance facilitated by our GAN-based synthetic-data-augmentation approach.

## 5. Discussion and Conclusions

The extensive application and promise shown by generative AI, especially in the domain of medicine and clinical settings, represent an evolution in healthcare analytics. Generative models, such as GANs, are particularly potent in scenarios where data are scarce, sensitive, or both, as is the case with many medical datasets [17]. They pave the way for synthesizing realistic patient data, enabling researchers to circumvent privacy constraints and enhance model training [18]. Furthermore, the potential of GANs extends beyond data augmentation. They have been employed for tasks such as medical image synthesis, anomaly detection in medical images, and even drug discovery [19,20]. Such advancements offer the potential to revolutionize diagnostic procedures, patient care, and treatment strategies. The integration of generative AI into medical applications heralds a new era where Machine Learning models not only aid in diagnosis, but also provide insights into novel therapeutic interventions and patient-specific care plans [21]. While these developments are promising, it is essential to approach with caution, ensuring rigorous validation and ethical considerations in the deployment of such AI-driven methodologies in healthcare.

In this sense, this work performed an evaluation of the Random Forests classifier on the augmented dataset, revealing compelling results, underscoring the effectiveness of GAN-generated synthetic data. The achieved AUC of 0.96 indicates a high degree of discrimination power, demonstrating the classifier’s ability to effectively distinguish between positive and negative instances. This high AUC value reflects the model’s capacity to accurately rank instances, which is crucial in medical diagnosis, where correctly identifying positive cases, i.e., individuals with diabetes, is of utmost importance.

Furthermore, the classifier exhibited an accuracy of 0.96, signifying the overall correctness of the predictions made by the model. This high accuracy suggests that the classifier performed exceptionally well on both positive and negative instances, contributing to its reliability as a diagnostic tool for diabetes classification. The precision value of 0.98 denotes the proportion of true positive predictions among all the instances classified as positive. This result highlights the classifier’s capability to minimize false positives, which is critical in medical diagnosis to avoid unnecessary treatments or interventions for healthy individuals.

The sensitivity, or recall, value of 0.94 indicates the proportion of true positive predictions among all actual positive instances. This metric represents the classifier’s ability to correctly identify individuals with diabetes, which is vital to ensuring early detection and timely medical intervention. Moreover, the specificity value of 0.98 denotes the proportion of true negative predictions among all actual negative instances. This demonstrates the classifier’s competence in accurately identifying individuals without diabetes, reducing the likelihood of misdiagnosing healthy individuals as diabetic.

The F1-score, which combines precision and recall, yielded a value of 0.96, emphasizing the overall robustness and balanced performance of the classifier. The F1-score considers both false positives and false negatives, making it an essential metric for evaluating classification models in imbalanced datasets, such as medical data.

This study illuminated the profound impact of Generative Adversarial Networks in augmenting the Random Forests classifier’s performance for diabetes classification, thereby addressing the data scarcity issue prevalent in medical datasets. The evaluation metrics underscore an improvement in accuracy, precision, sensitivity, and specificity, validating the merits of GAN-based data augmentation. Notwithstanding a slight dip in the AUC value, the overall enhancement in the classifier’s performance with a 30% increase in the dataset size manifests the potential of synthetic data in advancing healthcare analytics. The findings here echo the broader narrative of leveraging generative AI for developing robust Machine Learning models in healthcare, paving the path for accurate and timely diagnosis. Future explorations could extend into other medical domains and tackle ethical considerations incumbent in the deployment of AI-driven methodologies in healthcare.

It is crucial to highlight that the original dataset yielded an AUC of 0.98, a value that is 0.02 higher than that achieved with the synthetic data. Nonetheless, as previously emphasized, metrics such as specificity and accuracy witnessed a notable enhancement with the incorporation of synthetic data, culminating in an average performance improvement. Such an observation is particularly significant when one considers the overall uplift in the evaluation metrics alongside a 30% increase in the dataset size due to the addition of synthetic observations. These findings reiterate the delicate balance and nuanced decisions required when incorporating synthetic data, weighing the advantages of broader data representation against potential minor trade-offs in specific performance metrics.

The results obtained for the performance metrics underscore the significance of employing GAN-generated synthetic data in enhancing the Random Forests classifier’s performance for diabetes classification. The high AUC, accuracy, and precision values, coupled with substantial sensitivity and specificity, validate the utility of GAN-based augmentation in creating a more-comprehensive and -diverse dataset, ultimately contributing to more-accurate and -reliable diabetes diagnosis. These findings not only support the application of GANs for data augmentation, but also hold promise for broader applications in various medical domains, facilitating the development of robust and accurate Machine Learning models in healthcare.

This study not only demonstrated the effectiveness of GAN-generated synthetic data in enhancing the performance of the Random Forests classifier for diabetes classification, but also highlighted the potential of this approach to revolutionize Machine Learning models in healthcare. The comprehensive evaluation and significant performance improvements achieved through data augmentation underscore the practical utility of GANs in addressing data scarcity issues in medical datasets, a challenge that has long hindered the development of accurate predictive models.

Looking towards the future, several research directions emerge. Firstly, the application of generative models extends beyond diabetes classification. Exploring their use in other medical domains, such as cancer diagnosis, cardiovascular disease prediction, and rare disease identification, holds great promise. The ability to generate diverse and representative data can lead to more-accurate and -robust models across a spectrum of healthcare scenarios. Furthermore, the potential of Machine Learning and deep learning extends to various medical domains beyond diabetes classification. For instance, recent research highlighted the promise of deep learning approaches in improving the diagnosis of thyroid cancer. In a notable study, the authors concluded that deep learning could significantly enhance thyroid cancer diagnosis, although they emphasized the need for more-effective techniques, addressing data limitations, creating valid datasets, and establishing standard evaluation measures to realize this potential fully. They further suggested a collaborative approach, integrating deep learning algorithms with the expertise of radiologists, to enhance the accuracy and specificity of thyroid nodule diagnosis. This insight underscores the broader applicability and promise of Machine Learning in addressing diagnostic challenges across a range of medical conditions, reaffirming the importance of our findings in diabetes classification and suggesting a pathway for multidisciplinary and collaborative efforts in future research [22].

Secondly, ethical considerations remain paramount in the integration of AI-driven methodologies into healthcare. Future work should delve into the development of privacy-preserving generative models that adhere to stringent data protection regulations. Balancing the need for data-driven insights with patient privacy and confidentiality is an ongoing challenge that requires careful attention.

Furthermore, the continuous evolution of Machine Learning techniques and hardware capabilities opens avenues for real-time diagnostics and personalized treatment plans. The integration of generative models into telemedicine platforms and wearable healthcare devices can provide timely insights and recommendations to both patients and healthcare providers. A recent study extended the utility of deep learning into the domain of Point-Of-Interest (POI) recommendation systems, proposing a model that incorporates an attention mechanism to better integrate user-centric features and contextual information. Through evaluating on established datasets such as Yelp and Gowalla, the model exhibited significant improvement in precision and recall for POI recommendations by attentively factoring in users’ geographical patterns [23].

In conclusion, while this study focused on diabetes classification, its implications reach far beyond this single application. It exemplifies the potential of Generative Adversarial Networks to drive innovation in healthcare, addressing long-standing data challenges and ultimately improving patient care. The journey ahead involves interdisciplinary collaboration, ethical considerations, and a commitment to harnessing AI’s full potential to transform healthcare into a more-precise, -efficient, and -patient-centric endeavor.

## 6. Future Work

In the preceding sections, the utility of generative networks in augmenting clinical data was demonstrated, considering the inherent constraints associated with acquiring such data. However, in the context of Type 2 Diabetes (T2D), it is crucial to consider various other variables that affect this disease. As a prospective direction for future work, it is proposed to study patients’ extended health conditions using transformative approaches to gain a broader understanding of T2D, aiming to enhance the existing model. By employing Transformer-based models, a deeper insight into the multifaceted nature of T2D under varying health conditions can be achieved, which could potentially lead to better diagnostic and prognostic models. This could involve exploring relationships and patterns in the data that may help in better understanding the progression of T2D and its interaction with other health conditions. Additionally, investigating the potential of integrating more-comprehensive and -diverse data, including lifestyle, genetic, and other pertinent health metrics, could further refine the accuracy and reliability of predictive models, aiding in more-personalized and -effective patient care and management strategies for T2D.

## Figures and Tables

**Table 1:** Summary of patient data features: This table outlines the key features extracted from the patient dataset used in the study. It includes various demographic, physiological, and biochemical attributes that are crucial for the analysis and subsequent diagnosis of diabetes. The features range from basic demographic information such as age and sex to more-specialized medical indicators such as glucose levels and the Body Mass Index (BMI). The collection of these comprehensive features facilitates a multidimensional analysis aimed at creating an accurate and reliable diabetes classification model.

Feature	Description
Age	Age of the patient at the time the analysis was conducted
Sex	Sex of the patient (0 denotes male; 1 denotes female)
Education	Level of education achieved by the patient (1—elementary school; 2—secondary school; 3—high school; 4—Bachelor’s degree)
Weight	Weight of the patient, measured in kilograms
Height	Height of the patient, measured in centimeters
Waist	Waist circumference of the patient, measured in centimeters
Hip perimeter	Hip circumference of the patient, measured in centimeters
BMI	Body Mass Index, calculated based on the patient’s weight and height
WHR	Waist-to-Hip Ratio, calculated from the circumferences of the waist and hips
SBP	Systolic Blood Pressure, indicates the pressure in the blood vessels when the heart contracts
DBP	Diastolic Blood Pressure, indicates the pressure in the blood vessels during the relaxation phase of the heart’s cycle
Glucose	Level of glucose in the blood, measured in milligrams
MMO glucose	Level of glucose in the blood, measured in terms of a molar concentration
Insulin	Amount of insulin in the patient’s blood
HOMA	Homeostatic Model Assessment, an indicator of insulin resistance and beta-cell function
Cholesterol	Amount of cholesterol, a fat-like substance, in the patient’s cells
LDL	Level of Low-Density Lipoprotein in the patient’s body
HDL	Level of High-Density Lipoprotein in the patient’s body
TR	Level of Triglycerides, a type of lipid, in the patient’s body

**Table 2:** GAN configuration, Generator, and Discriminator settings: This table delineates the parameter settings chosen for both the Generator and the Discriminator components of the Generative Adversarial Network (GAN). The parameters were carefully selected to optimize the model’s performance in generating high-quality synthetic data that closely resemble the original dataset. The configurations represent a balanced trade-off between model complexity and the ability to capture the underlying data distribution, thus ensuring the effective generation of synthetic data for augmenting the original dataset.

Component	Configuration
Generator	Latent Dimension: 10
	Layers: 15 (ReLU), 30 (ReLU), 20 (Linear)
	Kernel Initializer: He Uniform
Discriminator	Layers: 25 (ReLU), 50 (ReLU), 1 (Sigmoid)
	Kernel Initializer: He Uniform
	Loss Function: Binary Cross-Entropy
	Optimizer: Adam
GAN	Discriminator Weights Non-Trainable
	Loss Function: Binary Cross-Entropy
	Optimizer: Adam
Training	Latent Dimension: 10
	Epochs: 10,000
	Batch Size: 128
	Evaluation Every: 200 Epochs

**Table 3:** Dataset size before and after synthetic data augmentation: This table outlines the total number of data samples in both the original dataset and the augmented dataset after the application of the GAN-based synthetic data generation. The augmentation aims to achieve a 30% increase in the dataset size, adhering to accepted data science practices for enhancing dataset diversity without compromising its integrity. The expanded dataset enables a more-robust evaluation of diabetes classification algorithms.

Dataset	Size
Original	1019
Real + synthetic data	1325

**Table 4:** Random Forests model metrics: AUC, specificity, sensitivity, accuracy, F1-score, and precision, calculated to evaluate the effectiveness of the Random Forests classifier. These metrics were computed based on the classification model trained using the expanded dataset, which includes both the original and GAN-generated synthetic data.

Metric	Value
AUC	0.96
Specificity	0.98
Sensitivity	0.94
Accuracy	0.96
F1-Score	0.96
Precision	0.98

**Table 5:** Random Forests model metrics presented by García et al. [5]: these metrics were computed based on the classification model trained using the original dataset.

Metric	Value
AUC	0.98
Specificity	0.93
Sensitivity	0.97
Accuracy	0.95
F1-Score	0.96
Precision	0.98

## Data Availability

Data are not available due to legal restrictions since they are part of a CONACyT project.
